# Standard versus low‐dose nab‐paclitaxel in previously treated patients with advanced non‐small cell lung cancer: A randomized phase II trial (JMTO LC14‐01)

**DOI:** 10.1002/cam4.5652

**Published:** 2023-02-21

**Authors:** Susumu Takeuchi, Kaoru Kubota, Shunichi Sugawara, Satoshi Teramukai, Rintaro Noro, Kei Fujikawa, Takashi Hirose, Shinji Atagi, Seigo Minami, Shinichiro Iida, Hiroshi Kuraishi, Tomoiki Aiba, Yuji Minegishi, Masaru Matsumoto, Masahiro Seike, Akihiko Gemma, Masaaki Kawahara, Japan‐Multinational Trial Organization (JMTO)

**Affiliations:** ^1^ Department of Pulmonary Medicine and Oncology, Graduate School of Medicine Nippon Medical School Tokyo Japan; ^2^ Department of Pulmonary Medicine Sendai Kousei Hospital Sendai Japan; ^3^ Department of Biostatistics Kyoto Prefectural University of Medicine Graduate School of Medical Science Kyoto Japan; ^4^ Department of Pulmonary Medicine and Oncology Nippon Medical School Tama Nagayama Hospital Tokyo Japan; ^5^ Department of Thoracic Oncology National Hospital Organization Kinki‐Chuo Chest Medical Center Osaka Japan; ^6^ Department of Respiratory Medicine Osaka Police Hospital Osaka Japan; ^7^ Department of Pulmonary Medicine Otemae Hospital Osaka Japan; ^8^ Department of Pulmonary Medicine Nagano Red Cross Hospital Nagano Japan; ^9^ Operations Office, The Japan‐Multinational Trial Organization Nagoya Japan

**Keywords:** low dose, nab‐paclitaxel, non‐small cell lung cancer, phase II trial, previously treated

## Abstract

**Background:**

Nab‐paclitaxel (nab‐PTX) has better transfer to tumor tissue than cremophor‐based paclitaxel. It suggests that the optimum dose of nab‐PTX might be lower than the dose and schedule that is widely used. We designed a randomized phase II trial to examine the clinical utility and safety of nab‐PTX in patients with previously treated advanced non‐small cell lung cancer (NSCLC).

**Methods:**

Patients were randomly allocated (1:1) to receive nab‐PTX monotherapy at 100 mg/m^2^ (group A) or 70 mg/m^2^ (group B). The primary endpoint was progression‐free survival (PFS). Secondary endpoints included overall survival (OS), objective response rate (ORR), and adverse events (AEs).

**Results:**

Finally, 81 patients were randomized. Similar results were observed in both groups for PFS (3.75 vs. 3.71 months), OS (13.50 vs. 16.13 months), or ORR (20.5% vs. 23.1%). The incidences of grade 3 or worse AEs were 57.5% in group A and 41.5% in group B. The proportion of serious side effects was 10.0% in group A and 4.9% in group B.

**Conclusion:**

Both standard dose and low dose of nab‐PTX monotherapy are active for previously treated NSCLC patients with better safety profile. Therefore, nab‐PTX 70 mg/m^2^ dose and schedule in the trial would be a reasonable option.

## Graphical abstract

In this phase 2 randomized clinical trial to examine activity and safety, and to determine optimum dose of nab‐PTX in patients with previously treated stage IIIB/IV or postoperative relapsed NSCLC, both 100 and 70 mg/m2 of nab‐PTX monotherapy were active in patients with previously treated advanced NSCLC. Since the 70 mg/m2 dose has a better safety profile and numerically favored median overall survival, 70 mg/m2 on days 1, 8, and 15 every 4 weeks would be the optimal dose and schedule for nab‐PTX during treatment of NSCLC.

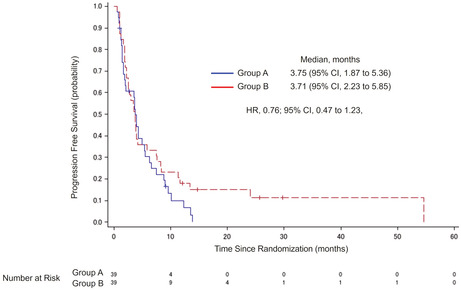

## 1 INTRODUCTION

Traditionally, the dose of anti‐cancer agents has been decided based on the incidence of dose‐limiting toxicity (DLT) in a phase I clinical trial. Ideally, however, the optimum dose should be decided based on efficacy and toxicity. This is because following a dose recommendation based on DLT alone, rather than the optimal dose (which may be lower) can increase toxicity, cost, and could immunocompromise patients without additional benefit. Defining the optimum dose of cytotoxic chemotherapy is therefore critical to improve overall survival (OS) and patient quality of life.

In chemotherapy for advanced NSCLC, administration of the optimal dose should improve outcomes. In the randomized phase II study of 1000 or 500 mg/m^2^ pemetrexed given to previously treated NSCLC patients, the objective response rate (ORR), median progression‐free survival (PFS), and OS were 18.5%, 3.0 and 16.0 months in the 500 mg/m^2^ arm 14.8%, 2.5 and 12.6 months in the 1000 mg/m^2^ arm. Although there was no statistically significant difference between these arms, it was noted that patients treated with the lower dose of 500 mg/m^2^ tended to have more favorable outcomes.^1^

In preclinical models of NSCLC, nab‐paclitaxel (nab‐PTX), an albumin bound and solvent free nanoparticle paclitaxel (PTX) formulation, has better transfer to tumor tissue than PTX. Compared with cremophor‐based PTX, nab‐PTX thus yielded higher mean intratumor and maximal circulating concentrations of free PTX in a xenograft model.^2^, ^3^ The recommended dose of nab‐PTX was subsequently decided based on DLT. A phase I/II trial of weekly nab‐PTX determined that 125 mg/m^2^ on Days 1, 8, and 15 of a 28‐day cycle was the maximum tolerated dose (MTD) in patients with stage IV NSCLC.^4^ A dose finding study of nab‐PTX followed by carboplatin AUC = 6 every 3 weeks in patients with advanced NSCLC found that ORR, PFS, and OS were 48%, 6.2 and 11.3 months following 100 mg/m^2^ of weekly nab‐PTX, and 36%, 6.4 and 15.0 months following 125 mg/m^2^ of weekly nab‐PTX.^5^ No clear dose response effects of nab‐PTX were observed in the study.

A weekly (Days 1, 8, and 15 every 28 days) 70 mg/m^2^ dose of paclitaxel is efficacious when combined with carboplatin.^6^ Considering its improved delivery to tumor cells, we estimated that the optimal dosing schedule for nab‐PTX would therefore be weekly administration of 70 mg/m^2^.

We designed a randomized phase II trial to determine the optimum dose, activity, and safety of weekly nab‐PTX (low dose 70 mg/m^2^ and standard 100 mg/m^2^) in patients with previously treated advanced NSCLC.

## 2 METHODS

### 2.1 Patient eligibility

The present trial, JMTO LC14‐01, was a multi‐institutional study and was designed as a randomized, phase II trial to be performed in wide area of Japan. The protocol was approved by the independent ethics committees of participating institutions. This trial was conducted in accordance with the Ethical Guidelines for Medical and Health Research involving Human Subjects, the Declaration of Helsinki, and the Clinical Trials Act in Japan. Written informed consent was obtained from all patients. This trial was registered with the UMIN Clinical Trials Registry (UMIN000016932) and the Japan Registry of Clinical Trials (jRCTs031180214).

For patients with previously treated stage IIIB‐IV or postoperative relapsed NSCLC, at least one of the previous treatment regimens had to have contained platinum‐based combination chemotherapy. In patients with *EGFR* gene mutation and *ALK* gene rearrangements, inhibitors of each kinase and platinum‐based combination chemotherapy had to have been received. Neo‐adjuvant or adjuvant chemotherapy was counted as one regimen if patients relapsed within 1 year of surgery or the last chemotherapy dose, respectively. Additional eligibility criteria consisted of measurable disease based on RECIST.ver1.1, age ≥ 20 years, Eastern Cooperative Oncology Group (ECOG) performance status (PS) of 0 or 1, adequate bone marrow, liver, and kidney function, life expectancy of more than 3 months. Written informed consent was provided from the patient. Key exclusion criteria included previous paclitaxel treatment, clinically symptomatic brain metastases, pleural effusion, ascites, or pericardial fluid that required drainage or other treatments, multiple active cancers, active infections, or complications, uncontrolled heart disease or diabetes mellitus, interstitial pneumonia upon chest X‐ray, psychiatric disease, pericardial effusion, intestinal obstruction or paralysis, and concurrent administration of oral or intravenous immunosuppressive agents including steroids. Pregnant or lactating women were excluded.

### 2.2 Study treatment and assessments

Patients were randomly allocated (one to one) to receive nab‐PTX at standard 100 mg/m^2^ (group A) over 30 min on Days 1, 8, and 15 every 4 weeks or nab‐PTX at low dose 70 mg/m^2^ (group B) over 30 min on Days 1, 8, and 15 every 4 weeks, using a minimization with stratification for pre‐chemotherapy regimen number (one or more than one), histology (squamous cell carcinoma or nonsquamous cell carcinoma), and medical institution. Both groups continued treatment until disease progression or the appearance of unacceptable toxicity was detected. If grade 3 or worse AEs were observed, the dose had to be reduced from 100 to 70 mg/m^2^ in group A and from 70 to 50 mg/m^2^ in group B.

### 2.3 Outcomes

The primary end point of the study was PFS (time from randomization to the date of objective disease progression or death from any cause in the absence of progression). Secondary end points included OS (time from the date of randomization to the date of death event by any cause), ORR (the proportion with radiologically confirmed partial response and complete response), according to Response Evaluation Criteria in Solid Tumors version 1.1, frequency and extent of AEs. AEs were graded in accordance with the National Cancer Institute Common Terminology Criteria for Adverse Events version 4.0. PFS and ORR were both assessed by blinded independent central review.

### 2.4 Statistical analysis

Based on the earlier trials for NSCLC patients with previously treated, the sample size was calculated to ensure the median PFS in each group exceeded 2 months. The assumptions of an expected median PFS of 3 months, 5% one‐sided significance level and 80% power, and an estimated of 2 years of enrollment and 1 year of follow‐up necessitated the inclusion of at least 37 patients in each arm. We planned to enroll 80 patients for the trial, accounting for dropout.

For each group, the cumulative PFS probability and median PFS were estimated using the Kaplan–Meier method. The 95% confidence interval (CI) for median PFS was estimated using the Brookmeyer and Crowley method. A test based on the maximum likelihood method for hazard assuming an exponential distribution was performed for the null hypothesis that the median PFS would be 2 months for each group. The hazard ratio (HR) was calculated using the Cox proportional hazards model. For each group, we estimated the ORR and the 95% CI. For reference, Fisher's exact test was used to make the null hypothesis that the ORRs between groups are equal. All statistical analysis was performed with SAS 9.4 software.

## 3 RESULTS

### 3.1 Patients

**FIGURE 1:**
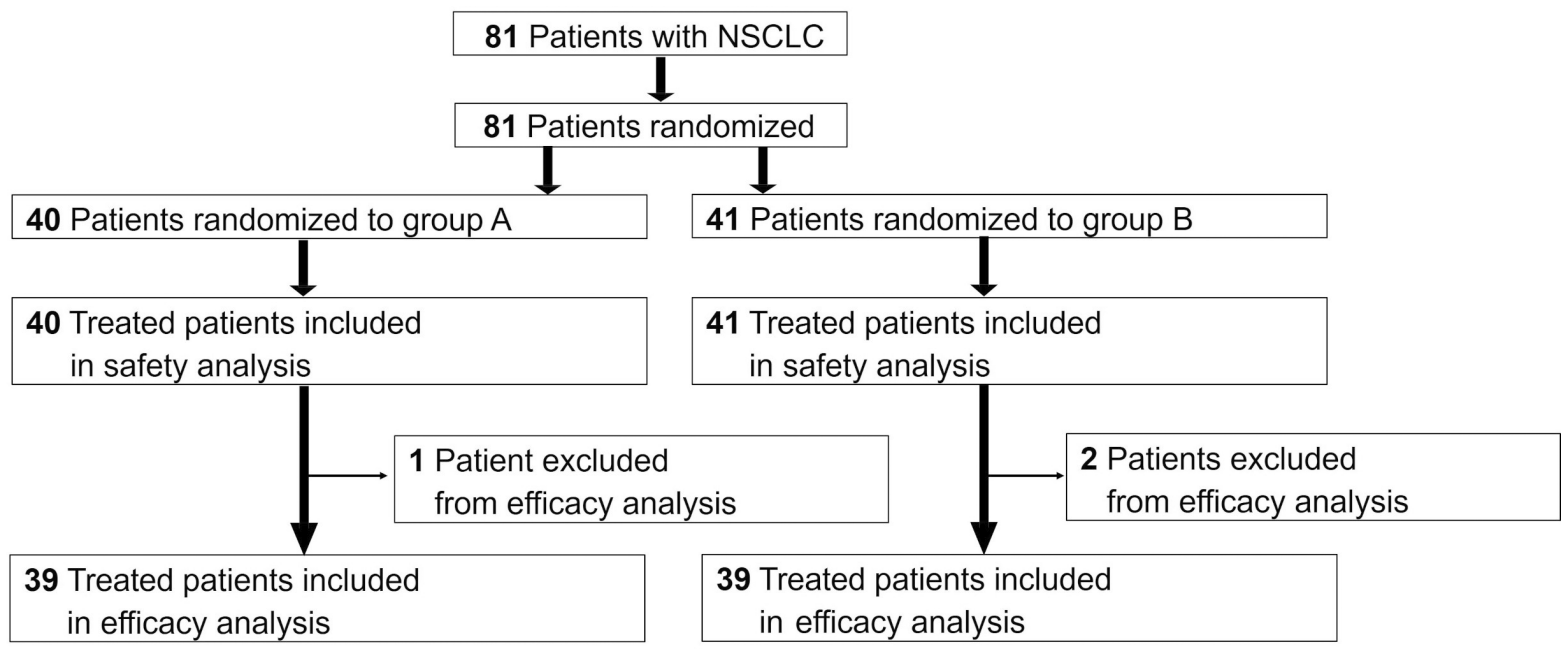
CONSORT Diagram of the study participants. Three patients were excluded from the efficacy analysis. However, these patients received study treatment and were included in the safety analysis. NSCLC, non‐small cell lung cancer.

Between May 2015 and May 2019, 81 patients were enrolled at 13 institutions in Japan and were randomly assigned to group A (40 patients) or group B (41 patients). One patient in group A was excluded from the efficacy analysis because their disease was at an inappropriate stage (stage IB), and 2 patients in group B were excluded from this analysis because of they received an EGFR inhibitor within 2 weeks of the study treatment, had elevated ALT, and had disease at an inappropriate stage (stage IIIA) (Figure 1). The baseline patient background and characteristics of the trial subjects were well balanced in the two groups (Table 1). For example, the median age was 67 years in group A (range 43–87) and 68 years in group B (range 38–79). Approximately 17% of patients in each group had *EGFR* mutant tumors. Patients with *EGFR* gene mutations and *ALK* gene rearrangements had been treated with at least one tyrosine kinase inhibitor. The number of patients enrolled for second‐line treatment was 17 (47.5%) in group A and 17 (46.3%) in group B. The number of patients enrolled for third‐line or subsequent treatment was 21 (52.5%) in group A and 22 (53.7%) in group B.

**TABLE 1:** Baseline demographic and clinical characteristics of the study patients.

Characteristic	Group A (*n* = 40)	Group B (*n* = 41)
Median age, years (range)	67 (43–87)	68 (38–79)
Gender		
Male	26 (65.0)	30 (73.2)
Female	14 (35.0)	11 (26.8)
ECOG performance status		
0	14 (35.0)	17 (41.5)
1	25 (62.5)	23 (56.1)
2	1 (2.5)	1 (2.4)
Clinical stage at study entry^a^		
III	5 (12.5)	3 (7.3)
IV	28 (70.0)	31 (75.6)
Postoperative recurrence	6 (15.0)	7 (17.1)
Smoking history		
Former or current smoker	27 (67.5)	34 (82.9)
Never smoker	13 (32.5)	7 (17.1)
Histology		
Squamous	10 (25.0)	11 (26.8)
Nonsquamous	30 (75.0)	30 (73.2)
*EGFR* gene mutation status		
Positive	7 (17.5)	7 (17.1)
Negative	26 (65.0)	32 (78.0)
Unknown	7 (17.5)	2 (4.9)
*ALK* gene rearrangement status		
Positive	1 (2.5)	1 (2.4)
Negative	30 (75.0)	36 (87.8)
Unknown	9 (22.5)	4 (9.8)
Number of previous cytotoxic chemotherapy		
1	19 (47.5)	19 (46.3)
2 or more	21 (52.5)	22 (53.7)

*Note*: With the exception of age, all values are number (%).

Abbreviations: *ALK*, Anaplastic lymphoma kinase; ECOG, Eastern Cooperative Oncology Group; *EGFR*, epidermal growth factor receptor gene.

a One patient was diagnosed as stage IB and excluded from efficacy analysis.

### 3.2 Therapeutic efficacy

**FIGURE 2:**
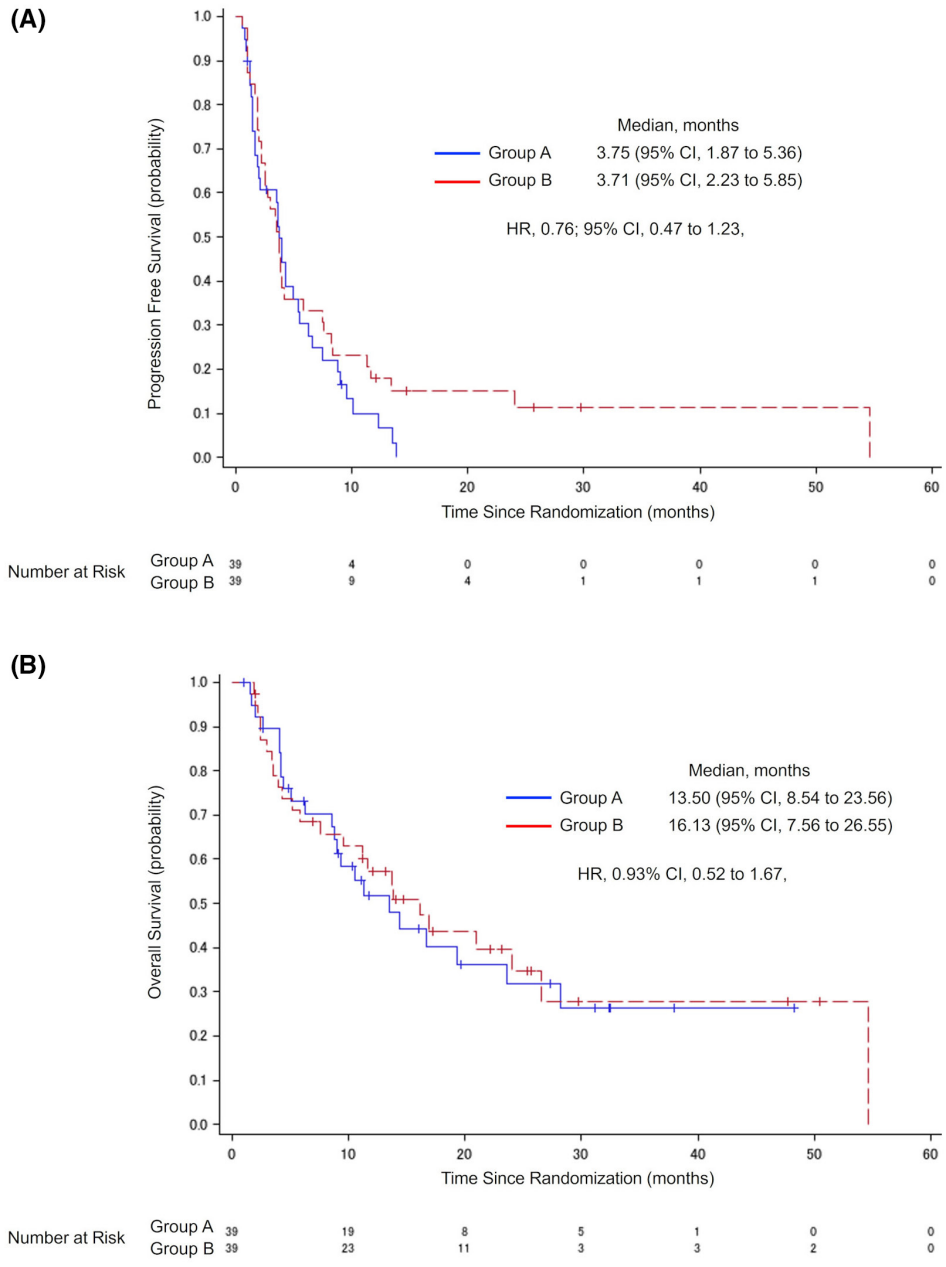
Investigator‐assessed progression‐free survival and overall survival. Kaplan–Meier plots for investigator‐assessed progression‐free survival (A) and overall survival (B) in the intention‐to‐treat (ITT) population. CI, confidence interval; HR, hazard ratio.

During the follow‐up period, events occurred in 36 (92.3%) of 39 patients in group A and 35 (89.7%) of 39 patients in group B regarding the primary endpoint. Median PFS was 3.75 months (95% CI, 1.87–5.36) in group A and 3.71 months (95% CI, 2.23–5.85) in group B (Figure 2A). The HR for group B to group A was 0.76 (95% CI, 0.47–1.23).

The results for OS are shown in Figure 2B. There were 23 events in group A and 24 in group B, with a median OS of 13.50 months (95% CI, 8.54–23.56) in group A and 16.13 months (95% CI, 7.56–26.55) in group B. The HR of group B to group A was 0.93 (95% CI, 0.52–1.67).

**FIGURE 3:**
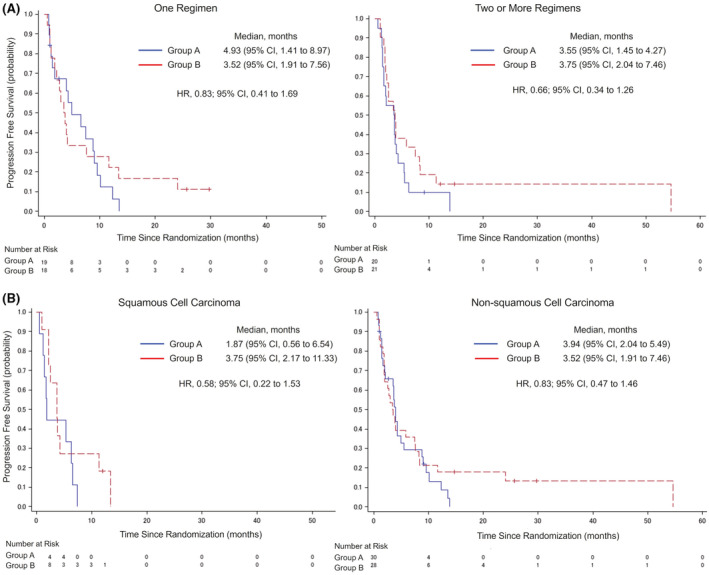
Investigator‐assessed progression‐free survival by number of prior cytotoxic chemotherapies and investigator‐assessed progression‐free survival by histologic subtype of tumor. Kaplan–Meier plots for subgroup analysis of investigator‐assessed progression‐free survival (A) and overall survival (B) in the intention‐to‐treat (ITT) population. Stratification factors for overall survival were the number of prior cytotoxic chemotherapy regimens (1 or ≧2), tumor histology (squamous or nonsquamous). CI, confidence interval; HR, hazard ratio.

The results of PFS and OS for subgroup analysis were consistent with those for the intention‐to‐treat population (Figure 3). Figure 3A shows the results of PFS by number of prior cytotoxic chemotherapies. Median PFS in patients who had received one regimen was 4·93 months (95% CI, 1.41–8.97) in group A and 3.52 months (95% CI, 1.91–7.56) in group B. Median PFS in patients who had received two or more regimens was 3.55 months (95% CI, 1.45–4.27) in group A and 3.75 months (95% CI, 2.04–7.46) in group B. The results of PFS by histologic subtype of tumor are shown in Figure 3B. In squamous cell carcinoma, the median PFS was 1.87 months (95% CI, 0.56–6.54) in group A and 3.75 months (95% CI, 2.17–11.33) in group B. The HR for group B to group A was 0.58 (95% CI, 0.22–1.53). In nonsquamous cell carcinoma, the median PFS was 3.94 months (95% CI, 2.04–5.49) in group A and 3.52 months (95% CI, 1.91–7.46) in group B. The HR for group B to group A was 0.83 (95% CI, 0.47–1.46).

The ORR was assessed for 39 patients who had measurable tumor lesions. The ORR was 20.5% (95% CI, 9.3–36.5) in group A and 23.1% (95% CI, 11.1–39.3%) in group B (Table 2). In addition, subgroup analysis of ORR based on histological subtype of tumor was evaluated. In squamous cell carcinoma, the response of group A was zero, there were 3 PR cases in group B, and ORR was 27.3% (95% CI, 6.0–61.0). In nonsquamous cell carcinoma, ORR was 26.7% (95% CI, 12.3–45.9) in group A and 21.4% (95% CI, 8.3–41.0) in group B. Table 3 shows the results of ORR by number of previous cytotoxic chemotherapies. Also, the result of OS subgroup analysis is shown in Figure S1.

**TABLE 2:** Response rates for enrolled patients and for histology‐based subsets.

Response rate^a^	Group A			Group B		
	*n*	%	95% CI	*n*	%	95% CI
Total enrolled patients	39			39		
Overall response	8	20.5	9.3 to 36.5	9	23.1	11.1 to 39.3
Complete response	0	0		0	0	
Partial response	8	20.5		9	23.1	
Stable disease	14	35.9		15	38.5	
Progressive disease	15	38.5		13	33.3	
Not evaluable	2	5.1		2	5.1	
Squamous subset	9			11		
Overall response	0	0	0.0 to 33.6	3	27.3	6.0 to 61.0
Complete response	0	0		0	0	
Partial response	0	0		3	27.3	
Stable disease	4	44.4		5	45.5	
Progressive disease	5	55.6		2	18.2	
Not evaluable	0	0		1	9.1	
Nonsquamous subset	30			28		
Overall response	8	26.7	12.3 to 45.9	6	21.4	8.3 to 41.0
Complete response	0	0		0	0	
Partial response	8	26.7		6	21.4	
Stable disease	10	33.3		10	35.7	
Progressive disease	10	33.3		11	39.3	
Not evaluable	2	6.7		1	3.6	

a Objective response by investigator review per Response Evaluation Criteria in solid tumors, version 1.1.

**TABLE 3:** Response rates by number of previous cytotoxic chemotherapies.

Response rate^a^	Group A			Group B		
	No.	%	95% CI	No.	%	95% CI
One regimen	19			18		
Overall response	5	26.3	9.1 to 51.2	4	22.2	6.4 to 47.6
Complete response	0	0		0	0	
Partial response	5	26.3		4	22.2	
Stable disease	6	31.6		8	44.4	
Progressive disease	6	31.6		5	27.8	
Not evaluable	2	10.5		1	5.6	
Two regimens or more	20			21		
Overall response	3	15.0	3.2 to 37.9	5	23.8	8.2 to 47.2
Complete response	0	0		0	0	
Partial response	3	15.0		5	23.8	
Stable disease	8	40.0		7	33.3	
Progressive disease	9	45.0		8	38.1	
Not evaluable	0	0		1	4.8	

a Objective response by investigator review per Response Evaluation Criteria in solid tumors, version 1.1.

### 3.3 Chemotherapy compliance status

Table 4 shows the compliance status of the study treatment. The median number of treatments received was 9.5 times (range, 2–45) in group A and 7.0 times (range, 1–36) in group B. The duration of treatments was 15.8 weeks (range, 2.9–60.9) in group A and 12.0 weeks (range, 2–57.7) in group B.

**TABLE 4:** Chemotherapy compliance status.

	Group A (*n* = 40)	Group B (*n* = 41)
Number of weekly treatments		
Median (range)	9.5 (2–45)	7 (1–36)
Duration of treatments		
Median (weeks) (range)	15.8 (2.9–60.9)	12.0 (2.0–57.7)
Cumulative dose		
Median (mg/m^2^) (range)	815 (200–4500)	490 (70–2520)
Dose intensity		
Median (mg/m^2^/week) (range)	65.0 (30.9–77.8)	48.3 (24.4–54.4)
Relative dose intensity		
Median (%) (range)	81.4 (38.6–103.7)	90.8 (44.4–103.7)
Number of patients with postponements		
*n* (%)	10 (25.0)	9 (22.0)
Number of patients with treatment skipped		
*n* (%)	22 (55.0)	11 (26.8)

The median cumulative nab‐PTX dose and dose intensity of group A was 815 mg/m^2^ (range, 200–4500) and 65.0 mg/m^2^/week (range, 30.9–77.8), respectively. On the contrary, the median cumulative nab‐PTX dose and dose intensity of group B was 490 mg/m^2^ (range, 70–2520) and 48.3 mg/m^2^/week (range, 24.4–54.4), respectively. The number of postponements was 10 (25.0%) in group A and 9 (22.0%) in group B. The main reason for the postponement was neutropenia, which occurred in 4 patients (40.0%) in group A and 1 patient (11.1%) in group B. Moreover, 22 (55.0%) treatments were skipped in group A and 11 (26.8%) were skipped in group B. The main reason for skipping treatment in both groups was due to neutropenia.

### 3.4 Safety

AEs occurred in 87.5% (35/40) of subjects in group A and 80.5% (33/41) in group B. Grade 3 or worse AEs occurred in 57.5% of patients (23 out of 40) in group A and 41.5% of patients (17 out of 41) in group B. Serious side effects developed in 10.0% of patients (4 out of 40) in group A and 4.9% of patients (2 out of 41) in group B (Table 5).

**TABLE 5:** Treatment‐related adverse events.

Treatment‐related adverse events	Group A (*n* = 40)			Group B (*n* = 41)		
	All grade	Grade 3	Grade 4	All grade	Grade 3	Grade 4/5^a^
Leukopenia	17 (42.5)	7 (17.5)	0	14 (34.1)	0	0
Neutropenia	22 (55.0)	11 (27.5)	4 (10.0)	11 (26.8)	5 (12.2)	1 (2.4)
Anemia	12 (30.0)	1 (2.5)	0	10 (24.4)	0	0
Thrombocytopenia	2 (5.0)	0	0	3 (7.3)	0	0
Febrile neutropenia	1 (2.5)	1 (2.5)	0	0	0	0
Fatigue	8 (20.0)	0	0	7 (17.1)	0	0
Anorexia	6 (15.0)	1 (2.5)	0	8 (19.5)	0	0
Interstitial lung disease	0	0	0	2 (4.9)	1 (2.4)	1 (2.4)^a^
Joint pain and muscle pain	3 (7.5)	1 (2.5)	0	2 (4.9)	0	0
Peripheral sensory neuropathy	4 (10.0)	0	0	6 (14.6)	0	0

*Note*: All values are number (%).

^a^ Grade 5 nab‐paclitaxel induced interstitial pneumonitis.

Among the AEs that occurred in 10 or more patients in group A were leukopenia in 17 patients (42.5%), neutropenia in 22 patients (55.0%), anemia in 13 patients (32.5%), and anorexia in 10 patients (25.0%). In group B, there was occurrence of leukopenia in 14 patients (34.1%), neutropenia in 11 patients (26.8%), and anemia in 10 patients (24.4%). All grades of peripheral sensory neuropathy occurred in 4 patients (10.0%) in group A and 6 patients (14.6%) in group B. These AEs were considered adverse drug reactions.

Serious AEs such as discontinuation of treatment occurred in 10.0% of patients (4 out of 40) in group A and 4.9% (2 out of 41) in group B. In group A, anemia, lung infection, hyperglycemia, and pneumothorax occurred in each of the 4 patients. In group B, interstitial lung disease (ILD) occurred in 2 patients. However, it was difficult to conclude that lung infection, hyperglycemia, and pneumothorax were treatment‐related adverse events. AEs leading to death occurred in 2.5% of patients (1 out of 40) in group A and 2.4% of patients (1 out of 41) in group B. The reason for the death of the group A patient was poor general condition with hyperuricemia, and suspicion of tumor progression. On the contrary, the death in group B was due to severe drug induced ILD.

## 4 DISCUSSION

The JMTO LC14‐01 phase II trial is the first randomized trial to evaluate optimal dose of nab‐PTX in previously treated patients with advanced NSCLC. Both 100 and 70 mg/m^2^ of nab‐PTX met the primary endpoint (PFS), and are efficacious in NSCLC. Both doses elicited similar PFS, OS, and ORR, and safety issues were less frequent and generally milder in patients in the low dose 70 mg/m^2^ arm. Although immunotherapy is the mainstream today based on the evidence from large randomized clinical trials, most patients with advanced NSCLC use cytotoxic agents when drug resistance develops.^7^, ^8^, ^9^, ^10^ A recent randomized phase III trial in patients with advanced NSCLC who had received prior treatments (the J‐AXEL study) demonstrated that nab‐PTX was more active than docetaxel in terms of PFS (4.2 vs. 3.4 months [HR 0.76, 95% CI, 0.63–0.92, *p* = 0.0042]) and ORR (29.9% vs. 15.4%, *p* = 0.0002). Although nab‐PTX was not superior in terms of OS, there was a trend of improved median OS with the drug (16.2 vs. 13.6 months, HR: 0.85, 95.2% CI: 0.68–1.07), and noninferiority of nab‐PTX to docetaxel was proven with less febrile neutropenia (2% vs. 22.1%) and a higher incidence of grade 3 or 4 peripheral sensory neuropathy (9.8% vs. 0.8%).^2^ The results of the present trial, especially with regard to the 70 mg/m^2^ dose, are similar to those of the J‐AXEL study, and we therefore suggest that this dose could be adopted as the standard therapy in previously treated patients with advanced NSCLC.

High‐dose medication is not always required to elicit clinical benefit. Indeed, greater efficacy at higher doses of chemotherapy was not observed in a solid tumor clinical trial.^11^ In our study, the median cumulative dose was 815 mg/m^2^ in group A and 490 mg/m^2^ in group B, but treatment skips were overwhelmingly higher in group A (55%). This suggests that 100 mg/m^2^ may exceed the tolerable dose in more than half of patients in group A.

A study of first‐line chemotherapy for advanced NSCLC showed that 100 mg/m^2^ of nab‐PTX plus carboplatin (CA031) elicited a higher ORR than cremophor‐based paclitaxel (33% vs. 25%, *p* = 0.005 [95% CI, 1.08–1.59]).^12^ In that trial, doses were delayed for 82% of patients in the nab‐PTX group and nab‐PTX dose reduction occurred in 46% of all treated patients. We therefore suggest that 70 mg/m^2^ of nab‐PTX also could be more suitable for first‐line chemotherapy with carboplatin.

Most toxicities were manageable with supportive care or appropriate dose reductions, and especially well tolerated in the 70 mg/m^2^ group, as patients experienced less severe drug‐related AEs. The low incidence of all grade peripheral sensory neuropathy and joint and muscle pain from both groups was probably due to the two‐week withdrawal period. Such a withdrawal period may also help sustain good quality of life for patients with advanced NSCLC. The death in group B was due to severe ILD, which indicates that nab‐PTX‐associated ILD can be a severe and potentially fatal adverse event. Patients with interstitial pneumonia (IP) are particularly at risk because they have a higher incidence of nab‐PTX‐related ILD.^13^ However, it has been reported that gastric cancer patients who received weekly nab‐PTX monotherapy develop less ILD (1.2%).^14^ Previous studies of nab‐PTX associated ILD reported that the incidence of ILD was 0.0%–8.3% in NSCLC patients with preexisting IP.^15^, ^16^, ^17^ In other words, nab‐PTX induced ILD is a rare adverse event, but is associated with a high mortality rate when it occurs. Because the onset of ILD is not related to dose level, physicians should be aware of the risk for ILD, even in patients treated with 70 mg/m^2^ dose of nab‐PTX.

The cost and supply of cancer drug are issues that should be considered in the healthcare system. The trial in our current study is in line with near‐equivalence concept.^18^ Although we did not conduct a formal cost–benefit analysis, it is clear from our data that a lower dose of nab‐PTX maintains the benefit for patients while reducing both treatment costs and toxicity.

Our trial had several limitations. First, pharmacokinetics or pharmacodynamics analysis was not conducted. However, pharmacokinetic data for nab‐PTX is similar for Japanese and non‐Japanese patients,^19^, ^20^, ^21^ and we can therefore extrapolate data from Japanese patients. Second, we did not collect data regarding post study treatment including ICIs because ICIs had not been approved in Japan when the present trial was started, which may have slightly biased the study towards a more favorable OS. Actually, there are possibilities that ICIs were used as post‐treatment in both groups. Third, the study population was small, and we did not have the statistical power to prove noninferiority of the lower dose.

In conclusion, both standard dose and low dose of nab‐PTX monotherapy are active for previously treated NSCLC patients with better safety profile. Therefore, nab‐PTX 70 mg/m^2^ dose and schedule in the trial would be a reasonable option in the treatment of NSCLC.

## AUTHOR CONTRIBUTIONS

**Susumu Takeuchi:** Conceptualization (lead); formal analysis (lead); investigation (lead); methodology (lead); writing – original draft (lead). **Kaoru Kubota:** Conceptualization (lead); formal analysis (lead); methodology (lead); project administration (lead); visualization (lead); writing – review and editing (lead). **Shunichi Sugawara:** Data curation (equal); investigation (equal); resources (equal); validation (equal); writing – review and editing (equal). **Satoshi Teramukai:** Conceptualization (equal); data curation (equal); formal analysis (lead); investigation (equal); methodology (lead); software (lead); validation (lead); writing – review and editing (equal). **Rintaro Noro:** Data curation (equal); investigation (equal); resources (equal); validation (equal); writing – review and editing (equal). **Kei Fujikawa:** Conceptualization (equal); formal analysis (equal); investigation (equal); methodology (equal); software (equal); validation (equal); writing – review and editing (equal). **Takashi Hirose:** Data curation (equal); investigation (equal); resources (equal); validation (equal); writing – review and editing (equal). **Shinji Atagi:** Data curation (equal); investigation (equal); resources (equal); validation (equal); writing – review and editing (equal). **Seigo Minami:** Data curation (equal); investigation (equal); resources (equal); validation (equal); writing – review and editing (equal). **Shinichiro Iida:** Data curation (equal); investigation (equal); resources (equal); validation (equal); writing – review and editing (equal). **Hiroshi Kuraishi:** Data curation (equal); investigation (equal); resources (equal); validation (equal); writing – review and editing (equal). **Tomoiki Aiba:** Data curation (equal); investigation (equal); resources (equal); validation (equal); writing – review and editing (equal). **Yuji Minegishi:** Data curation (equal); investigation (equal); resources (equal); validation (equal); writing – review and editing (equal). **Masaru Matsumoto:** Data curation (equal); investigation (equal); resources (equal); validation (equal); writing – review and editing (equal). **Masahiro Seike:** Data curation (equal); investigation (equal); resources (equal); validation (equal); writing – review and editing (equal). **Akihiko Gemma:** Data curation (equal); investigation (equal); resources (equal); supervision (lead); validation (equal); writing – review and editing (equal). **Masaaki Kawahara:** Conceptualization (lead); funding acquisition (lead); investigation (equal); methodology (equal); project administration (lead); supervision (lead); writing – review and editing (equal).

## FUNDING INFORMATION

This study was supported by Taiho Pharmaceutical Company Limited.

## CONFLICT OF INTEREST STATEMENT

Susumu Takeuchi reports receiving personal lecture fees from Taiho Pharmaceutical Company Limited. Kaoru Kubota received honoraria for lectures and consultation fee from Taiho. Shunichi Sugawara, Takashi Hirose, Yuji Minegishi, and Akihiko Gemma received honoraria for lectures from Taiho Pharmaceutical Company. Shinji Atagi and Masahiro Seike received grant and honoraria for lectures from Taiho Pharmaceutical Company. Satoshi Teramukai, Rintaro Noro, Kei Fujikawa, Seigo Minami, Shinichiro Iida, Hiroshi Kuraishi, Tomoiki Aiba, Masaru Matsumoto, and Masaaki Kawahara declared no conflict of interest.

## ETHICS STATEMENT

The protocol was approved by the independent ethics committees of participating institutions. This trial was conducted in accordance with the Ethical Guidelines for Medical and Health Research involving Human Subjects, the Declaration of Helsinki, and the Clinical Trials Act in Japan. Moreover, the written informed consent was obtained from all patients. This trial was registered with the UMIN Clinical Trials Registry (UMIN000016932) and the Japan Registry of Clinical Trials (jRCTs031180214).

## Supporting information


Figure S1.

## Data Availability

The data underlying this article will be shared on reasonable request to the corresponding author. All authors follow the FAIR principles (Findability, Accessibility, Interoperability, Reproducibility) for data access.
