# Supplementary figures and images for: Standard versus low‐dose nab‐paclitaxel in previously treated patients with advanced non‐small cell lung cancer: A randomized phase II trial (JMTO LC14‐01)
Source: Cancer Med. 2023 Feb 21;12(8):9133–43. doi: 10.1002/cam4.5652 (PMC10166935; doi:10.1002/cam4.5652)

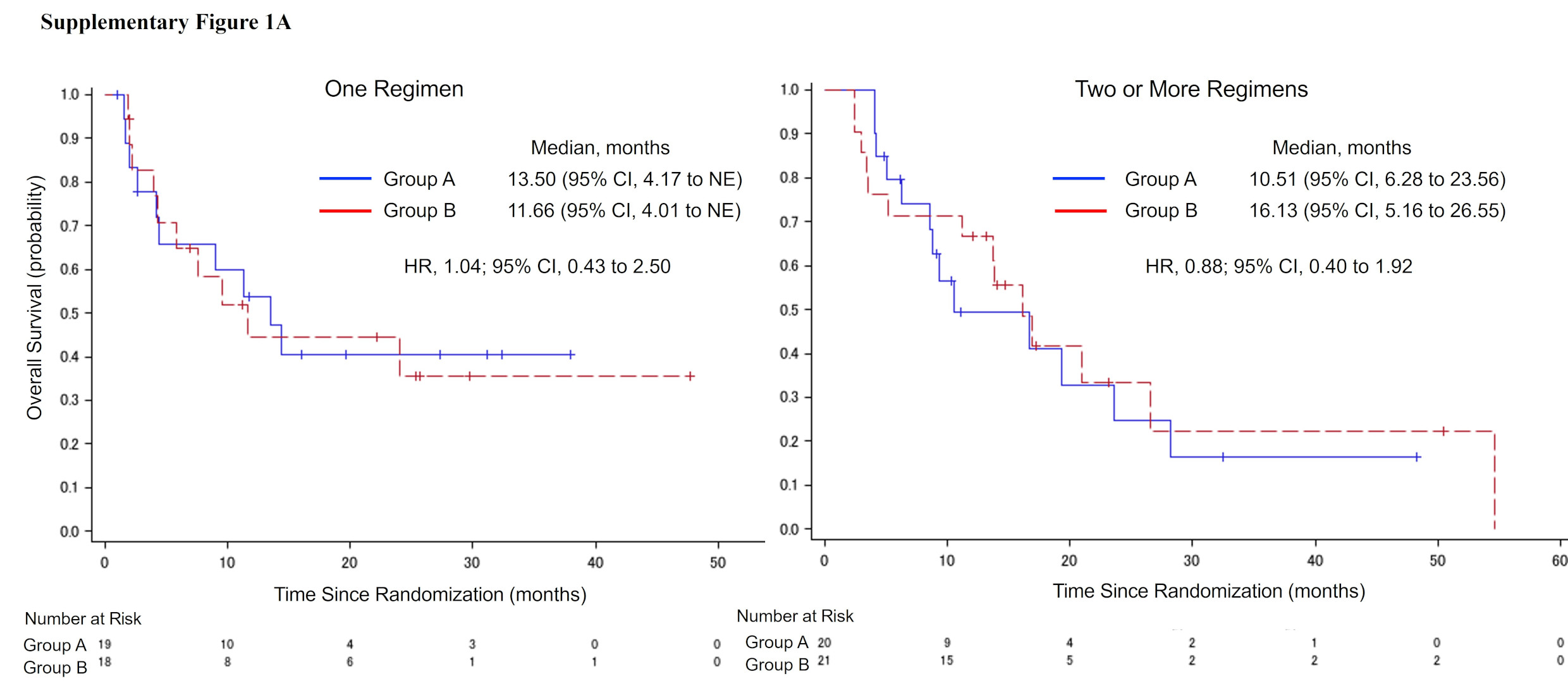

Supplement: Supplementary file 1 — Figure S1. [file CAM4-12-9133-s001.zip › CAM4_5652_Supplementary Figure1A.jpg]

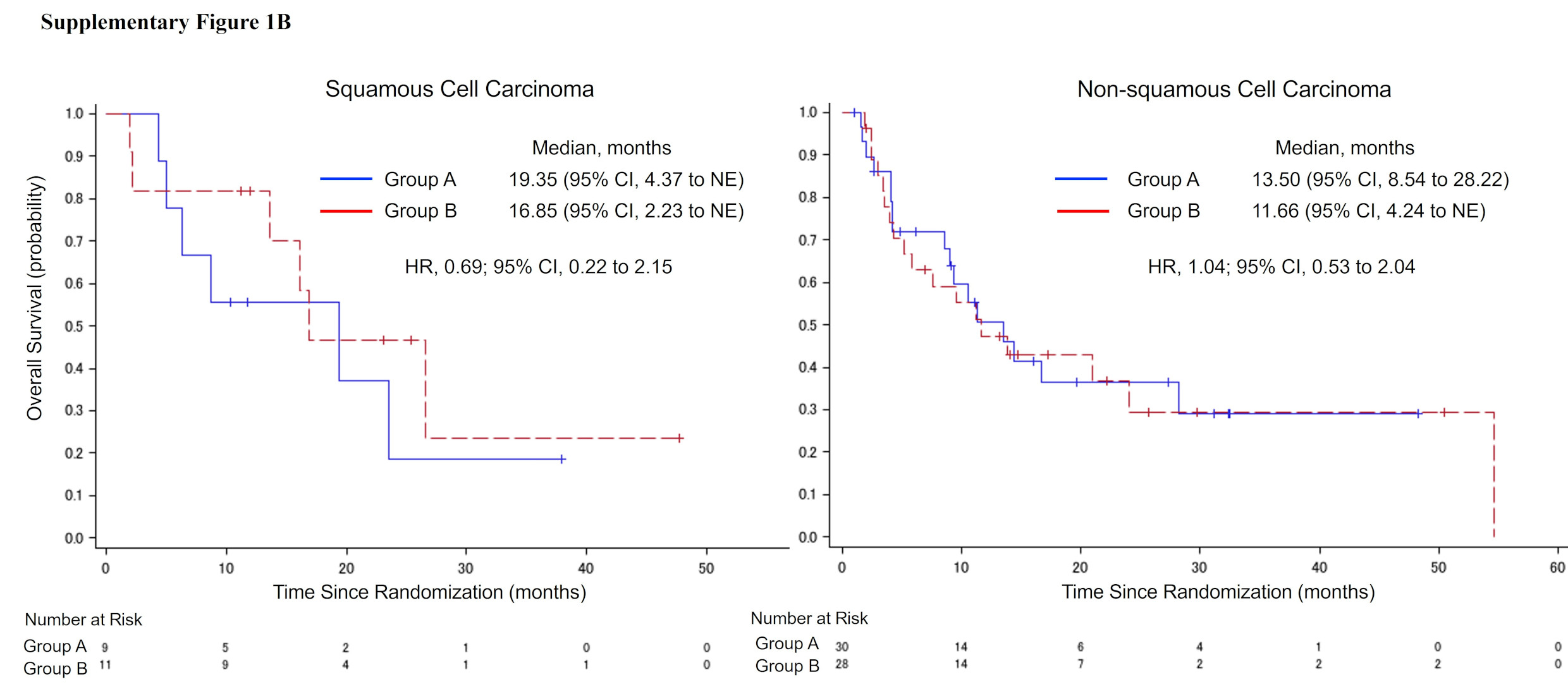

Supplement: Supplementary file 1 — Figure S1. [file CAM4-12-9133-s001.zip › CAM4_5652_Supplementary Figure1B.jpg]
